# Australian adolescents’ beliefs and help-seeking intentions towards peers experiencing symptoms of depression and alcohol misuse

**DOI:** 10.1186/s12889-017-4655-3

**Published:** 2017-08-16

**Authors:** D. I. Lubman, A. Cheetham, A. F. Jorm, B. J. Berridge, C. Wilson, F. Blee, L. Mckay-Brown, N. Allen, J. Proimos

**Affiliations:** 1 Turning Point, Eastern Health, Fitzroy, VIC Australia; 2 Eastern Health Clinical School, Monash University, Box Hill, VIC Australia; 3 Melbourne School of Population and Global Health, The University of Melbourne, Parkville, VIC Australia; 4 Illawarra Health and Medical Research Institute, Wollongong, NSW Australia; 5 School of Medicine, University of Wollongong, Wollongong, NSW Australia; 6 Travancore School, Victorian Department of Education and Training, Travancore, VIC Australia; 7 Melbourne Graduate School of Education, The University of Melbourne, Parkville, VIC Australia; 8 Melbourne School of Psychological Sciences, The University of Melbourne, Parkville, VIC Australia; 9 Department of Psychology, University of Oregon, Eugene, OR USA; 10 Victorian Department of Education and Training, Melbourne, VIC Australia

## Abstract

**Background:**

Many young people are reluctant to seek professional help for mental health problems, preferring to rely on their friends for support. It is therefore important to ensure that adolescents can identify signs of psychological distress in their peers, talk to them about these, and help them access appropriate services when necessary. The current study examined adolescents’ ability to recognise symptoms of depression and alcohol misuse, perceived barriers to help-seeking, and their intentions to encourage a peer to seek help from a range of informal and formal help sources.

**Method:**

The current study used baseline data from a randomised controlled trial of a school-based intervention that teaches adolescents how to overcome barriers to accessing professional help for mental health and substance use problems (*MAKINGtheLINK)*. Participants (*n* = 2456) were presented with two vignettes portraying depression and alcohol misuse, respectively, and were asked to identify the problems described. Participants provided data on their past help-seeking behaviour, confidence to help a peer, perceived barriers to help-seeking, and intentions to encourage a peer to seek help.

**Results:**

Health professionals were the main source of help that participants had relied on for depressive symptoms, followed by friends and parents. In contrast, friends were the main source of help that participants had relied on for alcohol and other drug problems, followed by health professionals and parents. Just over half of the sample correctly identified the problems described in the two vignettes, although the majority of participants were confident that they could talk to a peer and help them seek professional help if needed. Most agreed that the vignettes described problems that warranted professional help, however approximately half the sample was unsure or considered it unlikely that they would seek help if they experienced similar problems. For both disorders, participants were most likely to encourage a peer to seek help from their family, followed by formal help sources and friends.

**Conclusions:**

While the results point towards a greater willingness to approach formal help sources, particularly for depression, peers remain an important source of support for young people experiencing mental health and substance use problems.

## Background

Adolescents are often reluctant to seek professional help for mental health problems. In Australia, 12-17 year olds are more likely to approach informal sources (i.e., family and friends) than seek help from GPs, counselors, or mental health professionals [1–3], although family and friends may be approached less frequently by young people with moderate to severe levels of psychological distress [4–6]. While use of health services among adolescents experiencing mental illness has increased over the past two decades, a substantial minority (approximately 30%) do not report accessing any type of service [7]. Moreover, research among 16-24 year olds indicates that those experiencing substance use disorders may be the least likely to seek treatment [8]. These findings suggest that despite recent investments in early intervention services in Australia (e.g., *headspace,* a national youth mental health initiative dedicated to improving the wellbeing of young Australians) [9], many young people may not be receiving appropriate support for emerging mental health and substance use problems.

Improving the mental health literacy of adolescents and the individuals they turn to for support is likely to facilitate help-seeking [10]. Mental health literacy includes knowledge about disorders that aid in their recognition, management, and prevention, as well as attitudes that support problem recognition and appropriate help-seeking [11]. In 2006, it was estimated that 1% of the adult population in Australia had received training to improve their mental health literacy skills (Mental Health First Aid) [12], with more recent estimates indicating that this number has doubled over the last decade [13]. However, few interventions have focused on teaching adolescents how to help their peers. This is despite evidence that untrained adolescents are likely to have poor mental health literacy, and may lack the knowledge and skills to provide appropriate advice or support to others [14]. As such, there is likely to be considerable benefit in ensuring that adolescents are able to identify signs of psychological distress in their peers, approach and talk to them about these, address barriers to help-seeking, and help them access appropriate services when necessary [15]. In particular, there is a need to teach adolescents how to identify and assist with the most common mental health problems that affect their peers. Depressive and alcohol use disorders are of particular concern in this regard, as they typically begin in adolescence or young adulthood [16, 17], and account for a combined total of 50.5% of the global burden of disease attributed to mental illness [18].

Initial studies found that while approximately 50-60% of Australian adolescents could correctly identify the symptoms of depression from a vignette [19, 20], only 20-30% would encourage a peer to seek professional help for depression, with or without concurrent alcohol misuse (i.e., alcohol use disorders [1, 21]). More recently, studies have found evidence that recognition of depression and attitudes towards treatment may have been improved by campaigns to improve mental health literacy in young people, such as the *beyondblue* National Depression Initiative in Australia [22]. However, no research to date has examined whether there have been corresponding changes in the ways that adolescents intend to support their peers. In addition, few studies have examined adolescents’ ability to recognise signs of alcohol misuse independently, or examined the advice they give to peers experiencing alcohol-related problems.

There is also limited research examining gender differences in adolescents’ intentions towards helping their peers. This is of relevance given that studies have consistently found that females report fewer barriers to seeking help for their own mental health problems [23–27], as well as stronger intentions to seek help from a range of formal and informal sources [3, 23, 28–31]. However, in a pilot study of the program evaluated in the current paper, females reported greater confidence to help a peer experiencing substance use problems than males [32]. Moreover, in the only study to date examining gender differences in actual helping behaviours by adolescents, females were more likely to suggest and facilitate professional help-seeking as well as to personally help a close other experiencing mental health problems [33]. Together, this research suggests that adolescent females may be more likely to help their peers than males, for both depression and alcohol misuse.

The current study examined help-seeking for depression and alcohol misuse in a sample of 2464 Australian adolescents. We examined past help-seeking behaviour, confidence and intentions to help a peer, as well as perceived barriers to seeking professional help. We also sought to identify the sources of help that adolescents were most likely to recommend to peers experiencing depression or alcohol problems. Consistent with previous research, we expected that adolescents would be more likely to recommend seeking help from family and friends compared to formal help sources. In addition, it was hypothesised that females would be more likely to have sought help in the past, more likely to have helped a peer seek help, and would demonstrate more confidence and stronger intentions to help their peers, as well as fewer perceived barriers to seeking professional help.

## Method

### Design

The current study uses baseline data from a cluster randomised controlled trial of *MAKINGtheLINK,* a school-based intervention that teaches adolescents how to identify mental health and substance use problems in their peers, and helps them overcome barriers to accessing professional help [34].

### Participants

Twenty-two Victorian secondary schools were recruited for the study. Government (*n* = 14), Catholic (*n* = 5) and independent (*n* = 3) schools with between 75 and 250 Year 9 students were eligible for participation. Schools were recruited through a combination of emails, hard copy letters, and newsletters targeting secondary schools. Eligible participants were Year 9 students (aged 14-15 years). Schools were initially required to obtain consent forms signed by the students’ parent/guardian, however to increase participation rates a passive/opt-out consent process was later adopted. Under this process, students could make an informed choice as to whether they participated, however parents were informed in advance and could opt-out if they did not want their child involved [34]. Ethics approval was obtained by Monash University, the then Department of Education and Early Childhood Development Victoria (now Department of Education and Training), and the Catholic Education Office. Index of Community Socio-Educational Advantage (ICSEA) scores of the participating schools participating ranged from 913 to 1189, with an average of 1038. The ICSEA is a measure of educational advantage specifically developed for the Australian context, and was used to ensure that participating schools were representative in regard to levels of educational advantage (ICSEA values are scaled to a mean of 1000 and standard deviation of 100) [35]. In this context, educational advantage refers to both student-level (parents’ occupation, school education, and non-school education) and school-level (geographical location and the proportion of indigenous students catered for) factors.

### Procedure

The baseline assessment was group administered to students by a researcher during class time, online using SurveyMonkey©. Paper copies of the survey were also made available in the event of technical issues. The survey took approximately 30 min to complete and was programmed to maintain motivation and attention of the respondents. All students were provided with information on how to seek help for substance use, depression and other problems from a range of resources and services at the completion of the survey. Follow-up assessments were completed online during class 6 weeks after the initial survey and at 6 and 12 months (data from the follow-up assessments was not included in the current study).

### Measures

Past help-seeking behaviour was assessed using a simplified version of the Actual Help Seeking Questionnaire (AHSQ) [14], which was adapted to include substance use and mental health. We examined help-seeking for (i) stress and anxiety; (ii) depression; (iii) alcohol and other drug use; and (iv) another similar problem (e.g., bullying, problems at home, or relationship problems). For each problem reported, participants were asked who they sought help from (parent, friend, sibling, other family member, teacher, or health professional), and how helpful this was (rated on a 5-point scale from *1 = very helpful* to *5 = very unhelpful*; these responses were reverse-scored prior to analysis).

Participants were presented with two vignettes that described a young person with a mental disorder (Table 1). The first described ‘Sarah’ and depicted depression, while the second described ‘Samuel’ and depicted alcohol misuse. The depression vignette was written to satisfy the diagnostic criteria for major depression according to DSM-IV criteria (and align with DSM-5 diagnostic criteria), and was validated by surveys of mental health professionals [36]. A vignette depicting depression with comorbid alcohol misuse (based on DSM-IV alcohol abuse criteria) was also developed as part of the same study, which the current alcohol misuse vignette was based upon. These vignettes have been used extensively to assess problem recognition (one component of mental health literacy) in both adolescent and adult populations in Australia (for example, see [37–39], including in the National Survey of Mental Health Literacy and Stigma [40]). Free text responses were coded by a team of research assistants working to guidelines agreed upon by the team of Chief Investigators (academics with experience and publication records that include the coding of qualitative data). Coders met regularly to ensure consistency in coding and to discuss challenging examples. Coding was cross checked by other team members at regular intervals.

**Table 1:** Depression and alcohol misuse vignettes

Disorder	Vignette
Depression	*Sarah is your age and has been feeling unusually sad and miserable for the last few weeks. She is tired all the time and has trouble sleeping at night. Sarah doesn’t feel like eating, she has lost weight and has stopped hanging out with her friends. She can’t keep her mind on her studies and her marks have dropped. She puts off making any decisions and even day-to-day tasks seem too much for her.*
Alcohol misuse	*Samuel is a close friend the same age as you. Lately, he’s been getting smashed nearly every weekend at parties and doing things that are really embarrassing. The other week he got drunk and vomited. Some girls that were at the party posted pictures of him on Facebook with his head over a toilet. He’s also been getting aggro when he drinks and people aren’t inviting your group to parties any more.*

After being presented with the vignette, participants were asked (i) what, if anything, they thought was wrong with Sarah/Samuel; (ii) how confident they would be to approach Sarah/Samuel to talk about their concerns, and how confident they would be to help Sarah/Samuel seek professional help (both rated on a 4-point scale from *1 = not confident* to *4 = very confident*); (iii) whether they agreed that Sarah/Samuel needed professional help (rated on a 5 point scale from *1 = strongly disagree* to *5 = strongly agree*), and (iv) whether they would seek professional help if they were experiencing similar problems to Sarah/Samuel (rated on a 5 point scale from *1 = very unlikely* to *5 = very likely*).

Facilitation of help-seeking for a peer was assessed using a modified version of the General Help Seeking Questionnaire-Vignette [41]. After being presented with a study vignette (Table 1), participants rated how likely they would be to encourage a peer to seek help for alcohol misuse or depression from 15 different sources (e.g., boyfriend/girlfriend, friend, parent, teacher, GP) on a 5-point (*1 = very unlikely* to *5 = very likely*) Likert scale.

Help-seeking barriers were assessed using the brief version of the Barriers to Adolescents Seeking Help scale (BASH-B; [42]), which includes 11 items measuring beliefs that impede seeking professional psychological help. Responses were rated on a 4-point scale (1 = *Disagree* to 4 = *Agree*).

### Statistical analysis

All analyses were performed in SPSS version 22, with *p* < 0.05 indicating statistical significance. Chi-square analysis was used to analyse relationships between past help-seeking behaviour (i.e., past instances of seeking help for self and past instances of helping a friend) and the relationship between agreement that the problems described in the vignettes warranted professional help and participants’ intention to seek help if they were to experience similar problems. Non-parametric Friedman tests were used to analyse differences in participants’ intentions to recommend particular help sources, while Kruskal-Wallis tests were used to analyse differences in the perceived helpfulness of past help across the six sources that were assessed. Chi-square analyses were also used to analyse gender differences between help-seeking behaviour variables, while other differences between genders were analysed using paired samples t-tests.

## Results

### Demographics

In total, 2456 participants completed the baseline survey (1235 female, 1217 male; 4 did not report gender). Participants were 14.9 years old on average (SD = 0.45 years) at the time of the assessment. The average response rate per school was 77.4%. In total, 84.2% of participants were born in Australia; the next most common country of birth was New Zealand (1.9%), followed by the United Kingdom (1.4%), India (1.1%) and China (1.0%). All other countries of birth were reported by less than 1% of participants. The main language spoken at home was English (84.1%), followed by Vietnamese (2.8%), Arabic (2.4%), and Turkish (1.2%).

### Help-seeking behaviour

Almost half of the sample (1135 participants; 46.2%) reported seeking help for self-reported symptoms of stress and anxiety, depression, alcohol, drugs, and/or another similar problem. Stress and anxiety was the most common problem, followed by “another similar problem”, depression, and alcohol and other drugs respectively (AOD; Table 2). “Similar problems” included bullying (*n* = 298), problems at home (*n* = 246), school problems (*n* = 26), relationship problems (*n* = 166), insomnia (*n* = 5), and suicidal thoughts or attempts (*n* = 15). The majority of adolescents who had sought help (70-80%) reported doing so within the last 12 months, for each problem type.

**Table 2:** Main source of help for mental health and substance use problems

	Parent		Friend		Sibling		Other relative		Teacher		Health professional		Total	
Stress/anxiety	284	34.5%	182	22.1%	40	4.9%	16	1.9%	25	3.0%	276	33.5%	823	100%
Depression	107	24.7%	122	28.2%	12	2.8%	15	3.5%	5	1.2%	172	39.7%	433	100%
AOD	7	10.0%	33	47.1%	5	7.1%	6	8.6%	4	5.7%	15	21.4%	70	100%
Other	195	29.1%	186	27.8%	24	3.6%	24	3.6%	65	9.7%	175	26.2%	669	100%

Parents, friends, and health professionals were the main sources of help, although help-seeking behaviour varied by problem type. For stress and anxiety, more adolescents had sought help from parents and health professionals than from friends. For depression, more adolescents had sought help from health professionals than from parents and friends. For alcohol and other drug-related problems, more adolescents had sought help from friends than from parents or health professionals. Siblings, other relatives, and teachers were used less frequently for support.

Health professionals and teachers were generally perceived as most helpful, although average ratings were low (typically between ‘unhelpful’ and ‘neither helpful nor unhelpful’). Parents and friends were perceived as least helpful, with an average rating between ‘very unhelpful’ and ‘unhelpful’ (Table 3). There were significant differences between helpfulness ratings across the six sources of help for stress/anxiety (H(2) = 48.73, *p* < 0.001), depression (H(2) = 23.77, *p* < 0.001), and other problems (H(2) = 40.03, *p* < 0.001). Dunn-Bonferroni post-hoc tests found that health professionals were rated as significantly more helpful than parents and friends for stress/anxiety, depression, and other problems (all *p* values <0.01). Teachers were also rated as significantly more helpful than parents for other similar problems (*p* < 0.001). No differences in helpfulness were found in relation to alcohol and other drugs (H(2) = 6.40, *p* = 0.269), which is likely due to a lack of statistical power as fewer participants reported these problems.

**Table 3:** Perceived helpfulness of help sources (1 *= very unhelpful* to *5 = very helpful*)

	Parent		Friend		Sibling		Other relative		Teacher		Health professional	
	Mean	SD	Mean	SD	Mean	SD	Mean	SD	Mean	SD	Mean	SD
Stress/anxiety	1.62	.763	1.86	.933	2.05	1.218	1.56	.727	1.88	.927	2.17	1.017
Depression	1.81	.982	1.94	1.007	2.08	1.084	2.20	1.014	3.40	1.517	2.31	1.115
AOD	1.57	.787	1.91	.980	2.40	.894	2.00	1.095	2.25	.957	2.53	1.246
Other	1.64	.742	1.85	.929	1.88	.612	1.67	.637	2.26	1.108	2.17	.965

A further 1257 participants (51.2%) reported encouraging a friend to seek help for stress, anxiety, alcohol, drugs, or another similar problem. Adolescents were more likely to encourage a friend to seek help if they had sought help themselves (71.0% of those who had sought help had encouraged a friend compared to 43.0% of those who had not; X^2^ = 175.623, *p* < 0.001).

### Mental health literacy and help-seeking intentions

Over half of participants were able to correctly identify depression from the vignettes (Table 4). A further third (31.2%) either identified a different mental health condition, a physical health problem, provided a non-clinical description of the problem (e.g., ‘she’s sad’ or ‘stressed out’), or described a negative event that may have acted as a trigger. Of the remainder, 6.8% responded ‘don’t know’ or said that nothing was wrong (e.g., what Sarah was experiencing was ‘just part of adolescence’), while 2.0% did not answer the question or provided an illegible or judgemental response (e.g., ‘she’s just weak’).

**Table 4:** Responses to the depression and alcohol misuse vignettes

Depression (Sarah)	*n*	%	Alcohol misuse (Samuel)	*n*	%
Depression	1472	59.7%	Alcohol/drug problem	1287	52.3%
Other mental health condition	97	3.9%	Other mental health condition	222	9.0%
Non-clinical mood/behavioural descriptors	325	13.2%	Lack of knowledge/poor decision making	71	2.9%
Alcohol/drugs	76	3.1%	Poor coping/problem management	165	6.7%
Negative experience trigger	258	10.4%	Peer/social context	151	6.1%
Physical health problem	15	0.6%	Negative experience trigger	121	4.9%
Don’t know/unable to identify a problem	133	5.4%	Don’t know/unable to identify a problem	214	8.7%
Nothing/it’s normal/just adolescence	34	1.4%	Nothing/it’s normal/just adolescence	109	4.4%
Weak/lazy	11	0.4%	Weak/stupid/attention-seeking	41	1.7%
Missing	43	1.6%	Missing	82	3.3%

A slightly smaller proportion correctly identified alcohol misuse from the vignettes. Again, approximately a third (29.6%) provided alternative answers, identifying other mental health conditions, poor decision-making or problem management skills, the influence of peers, or describing a negative triggering event. Of the remainder, 13.1% responded ‘don’t know’ or that nothing was wrong (e.g., Samuel was just being ‘a normal teenager’ or ‘trying to have fun’), while 5.0% did not answer or provided an illegible or judgemental response (e.g., ‘he’s an idiot’).

Most adolescents were at least slightly confident that they could talk to Sarah and Samuel about their concerns, and help them seek professional help (Table 5). Confidence was higher regarding seeking professional help, with approximately 20% of participants ‘very confident’ and only 5% ‘not confident.’

**Table 5:** Confidence to talk to a peer and help them seek professional help

How confident would you be to…	Very confident		Confident		Slightly confident		Not confident	
Talk to Sarah about your concerns? ^a^	284	11.6%	905	36.8%	999	40.6%	260	10.6%
Help Sarah seek professional help? ^a^	598	24.3%	1165	47.4%	584	23. 8%	95	3.9%
Talk to Samuel about your concerns? ^b^	281	11.4%	772	31.4%	939	38.2%	425	17.3%
Help Samuel seek professional help? ^b^	466	19.0%	1087	44.3%	683	27.8%	172	7.0%

^a^Depression; ^b^Alcohol misuse

The majority of participants agreed that both Sarah and Samuel need professional help (Table 6). However, approximately 20% of respondents to each statement neither agreed nor disagreed, while approximately 5% disagreed.

**Table 6:** Beliefs regarding the need for professional help

	Strongly agree		Agree		Neither agree nor disagree		Disagree		Strongly disagree	
Sarah needs professional help ^a^	651	26.5%	1213	49.4%	473	19.3%	91	3.7%	20	0.8%
Samuel needs professional help ^b^	759	30.9%	1056	43.0%	430	17. 5%	116	4.7%	51	2.1%

^a^ Depression; ^b^ Alcohol misuse

When adolescents were asked if they would seek professional help if they had a similar problem, approximately 40% said it was likely in response to both vignettes, 30% were unsure, and 20-30% said it was unlikely (Table 7). Participants who agreed or strongly agreed that Samuel needed professional help were more likely to seek help if they had a similar problem (Chi-squared = 288.88, *p* < 0.05). Similarly, participants who agreed or strongly agreed that Sarah needed professional help were more likely to seek help for a similar problem (Chi-squared = 109.21, *p* < 0.05).

**Table 7:** Future help-seeking intentions

Would you seek professional help…	Very likely		Likely		Not sure		Unlikely		Very Unlikely	
If you had a problem like Sarah’s ^a^	342	13.9%	667	27.7%	736	30.0%	423	17.2%	275	11. 6%
If you had a problem like Samuel’s ^b^	379	15. 4%	728	29.6%	767	31.2%	300	12.2%	235	9.5%

^a^Depression; ^b^Alcohol misuse

Participants tended to agree with similar barriers to seeking help for depression and alcohol problems (Tables 8 and 9). For both vignettes, the main barriers were self-reliance, followed by embarrassment. A smaller percentage agreed with barriers regarding time and money. The barrier that participants were least likely to agree with was ‘nothing will change the problems that I have.’

**Table 8:** Barriers to seeking professional help for depression

	Agree		Somewhat agree		Somewhat disagree		Disagree	
I would solve my problem myself	546	22.2%	1149	46.6%	558	22.7%	189	7.7%
I think I should work out my own problems	573	23.3%	1106	44.9%	593	24.1%	168	6.8%
I’d be too embarrassed to talk to a counsellor	591	24.0%	809	32.8%	594	24.1%	446	18.1%
Adults can’t understand adolescent problems	337	13.7%	705	28.6%	783	31.8%	611	24.8%
Even if I wanted to, I wouldn’t have time to see a counsellor	214	8.7%	580	23.5%	890	36.1%	754	30.6%
A counsellor might make me do what I don’t want to	469	19.0%	876	35.6%	682	27.7%	411	16.7%
I wouldn’t want my family to know I was seeing a counsellor	613	24.9%	654	26.5%	593	24.1%	579	23.5%
I couldn’t afford counselling	294	11.9%	508	20.6%	741	30.1%	896	36.4%
Nothing will change the problems I have	176	7.1%	334	13.6%	794	32.2%	1132	45.9%
If I go to counselling, I might find out I’m crazy	213	8.6%	392	15.9%	778	31.6%	1058	42.9%
If I went for help, the counsellor would not keep my secret	321	13.0%	478	19.4%	616	25.0%	1022	41.5%

**Table 9:** Barriers to seeking professional help for alcohol misuse

	Agree		Somewhat agree		Somewhat disagree		Disagree	
I would solve my problem myself	523	21.2%	967	39.3%	621	25.2%	295	12.0%
I think I should work out my own problems	511	20.7%	1051	42.7%	585	23.7%	256	10.4%
I’d be too embarrassed to talk to a counsellor	445	18.1%	809	32.8%	696	28.3%	452	18.3%
Adults can’t understand adolescent problems	315	12.8%	573	23.3%	837	34.0%	673	27.3%
Even if I wanted to, I wouldn’t have time to see a counsellor	213	8.6%	534	21.7%	868	35.2%	790	32.1%
A counsellor might make me do what I don’t want to	404	16.4%	811	32.9%	668	27.1%	517	21.0%
I wouldn’t want my family to know I was seeing a counsellor	532	21.6%	698	28.3%	605	24.6%	569	23.1%
I couldn’t afford counselling	241	9.8%	490	19.9%	731	29.7%	941	38.2%
Nothing will change the problems I have	139	5.6%	310	12.6%	835	33.9%	1118	45.4%
If I go to counselling, I might find out I’m crazy	192	7.8%	393	16.0%	778	31.6%	1043	42.3%
If I went for help, the counsellor would not keep my secret	302	12.3%	548	22.2%	683	27.7%	871	35.4%

Table 10 reports the proportion of adolescents who were very likely or likely to encourage a peer to seek help from each source. Average ratings were calculated for each help source, ranging from 5 (very likely) to 1 (very unlikely). A significant difference between help sources was evident for depression (χ^2^ = 3364.37, *p* < 0.001), with adolescents most likely to encourage Sarah to seek help from her family (M = 4.19, SD = 0.85), followed by formal sources (M = 3.68, SD = 0.68), peers (M = 3.30, SD = 0.87) and the internet (M = 2.43, SD = 1.00). Similarly, there was a significant difference between help sources for alcohol misuse (χ^2^ = 2267.31, *p* < 0.001), with adolescents most likely to encourage Samuel to seek help from his family (M = 3.86, SD = 1.02), followed by formal sources (M = 3.66, SD = 0.79), peers (M = 3.13, SD = 0.95) and the internet (M = 2.49, SD = 1.04). Post-hoc tests indicated that all pairwise comparisons between help sources were significant at the *p* < 0.001 level, for both vignette conditions.

**Table 10:** Percentage likely to encourage a peer to seek help from each help source

Depression (Sarah)	n %		Alcohol misuse (Samuel)	n %	
Her mother	2124	86.2%	School counsellor	1793	72.8%
School counsellor	1998	81.1%	Mental health professional outside of school	1757	71.3%
Her father	1984	80.5%	His father	1745	70.8%
Another relative or family member	1932	78.4%	His mother	1712	69.5%
Mental health professional outside of school	1905	77.3%	Alcohol and drug worker	1685	68.4%
Phone help line (e.g., Lifeline, Kids Helpline)	1632	66.2%	Another relative or family member	1647	66.8%
Doctor/GP	1468	59.6%	Phone help line (e.g., Lifeline, Kids Helpline)	1487	60.4%
Teacher	1461	59.3%	Doctor/GP	1475	59.9%
A friend (not related to Sarah)	1304	52.9%	A friend (not related to Samuel)	1169	47.4%
Her boyfriend or girlfriend	1132	45.9%	His boyfriend or girlfriend	929	37.7%
Information from a reliable internet website	732	29.7%	Teacher	890	36.1%
Alcohol and drug worker	560	22.7%	Information from a reliable internet website	749	30.4%
Information from an internet chat room or blog	312	12.7%	Information from an internet chat room or blog	359	14.6%
I would not encourage Sarah to seek help from anyone	175	7.1%	I would not encourage Samuel to seek help from anyone	240	9.7%

### Gender differences

Females were significantly more likely to seek help than males (57.8% compared to 37.0%; *χ* ^2^ = 102.486, *p* < 0.001), and to have helped a friend seek help in the past (66.9% compared to 44.3%; *χ* ^2^ = 113.615, *p* < 0.001). The type of help source also varied by gender for stress and anxiety (*χ* ^2^ = 13.346, *p* = 0.020), depression, (*χ* ^2^ = 16.687, *p* = 0.005), and another similar problem (*χ* ^2^ = 14.117, *p* = 0.015), with females more likely to rely on health professionals as their main source of help. Females were significantly more confident that they could help a peer experiencing symptoms of depression to seek professional help, but significantly less confident that they could help a peer experiencing alcohol misuse. Other differences in confidence were not significant (Table 11).

**Table 11:** Gender differences in confidence to help a peer

How confident would you be to…	Males		Females		t(df)	*p*
	Mean	SD	Mean	SD		
Talk to Sarah about your concerns? ^a^	2.48	.874	2.52	.789	−1.194 (2.442)	0.233
Help Sarah seek professional help? ^a^	2.85	.836	3.01	.746	−5.008 (2437)	<0.001
Talk to Samuel about your concerns? ^b^	2.49	.950	2.26	.845	6.228 (2413)	<0.001
Help Samuel seek professional help? ^b^	2.77	.875	2.76	.807	0.331 (2404)	0.741

^a^Depression; ^b^Alcohol misuse

There were significant gender differences in regard to participants’ agreement that the problems described in the vignette warranted professional help. For depression, agreement was higher amongst females (M = 4.04, SD = 0.78) than males (M = 3.90, SD = 0.86; *t =* −4.302 [df = 2443], *p <* 0.001). Similarly, for alcohol misuse, agreement was higher amongst females (M = 4.06, SD = 0.84) than males (M = 3.89, SD = 1.02; *t =* −4.590 [df = 2408], *p <* 0.001). While there were no gender differences in the likelihood of seeking help for similar problems, females perceived greater barriers to seeking help than males. This was evident in regard to depression (females M = 2.38, SD = 0.58; males M = 2.30, SD = 0.58; *t =* −3.368 [df = 2437], *p =* 0.001) as well as alcohol misuse (females M = 2.31, SD = 0.62; males M = 2.23, SD = 0.62; *t =* −3.111 [df = 2404], *p =* 0.002). Finally, females were more likely to encourage a peer to seek help from formal and internet sources for depression, and from family and formal sources for alcohol misuse. There were no other gender differences in help-seeking intentions (Table 12).

**Table 12:** Gender differences in likelihood of encouraging a peer to seek help

Help source	Male		Female			
	Mean	SD	Mean	SD	*t*(df)	*p*
Depression						
*Peer*	3.29	0.92	3.31	0.81	−0.669 (2437)	.504
*Family*	4.19	0.90	4.20	0.77	−0.171(2439)	.864
*Formal*	3.59	0.74	3.77	0.61	−6.445(2436)	.000
*Internet*	2.36	1.01	2.49	0.98	−3.283(2436)	.001
Alcohol misuse						
*Peer*	3.13	0.99	3.13	0.91	−0.084(2406)	.933
*Family*	3.79	1.13	3.94	0.89	−3.590(2406)	.000
*Formal*	3.51	0.85	3.81	0.69	−9.563(2406)	.000
*Internet*	2.46	1.07	2.53	1.02	−1.597(2406)	.110

## Discussion

The current study provides insight into the help-seeking behaviours of Australian adolescents, as well as their beliefs and intentions towards peers experiencing depression and alcohol use problems. Approximately half of the sample had sought help for mental health or substance use problems in the past, with parents, friends, and health professionals the three main sources of support. Our findings contrast with the results of some earlier studies, in which adolescents tended to approach formal help sources less frequently than family and friends [2, 3], although they are consistent with previous findings amongst adolescents who experience more severe symptoms of psychological distress [4–6]. In particular, we found that health professionals were the primary source of support for adolescents who had sought help for depression. This is not unexpected, given that young people are increasingly seeking professional help for mental health problems [7], and considering that public recognition of depression and beliefs about treatment has improved over the past two decades, likely due in part to the influence of the *beyondblue* Australian National Depression Initiative [22]. However, it is important to note that while health professionals were consistently rated as more helpful than family or friends, their overall helpfulness rating was low (with an average between ‘unhelpful’ and ‘neither helpful nor unhelpful’). This may be a concern if participants’ have negative past experiences that lead them to be unwilling to seek professional help in the future.

While close to 20% of the sample had sought help for depression, only 3% had sought help for alcohol and other drugs. As the prevalence of substance use disorders amongst 16-24 year olds is approximately double that of affective disorders (Reavley et al., [8]), many young people may not be seeking help for emerging substance use problems in early adolescence. Moreover, those that did seek help relied primarily on friends for support, with a smaller proportion approaching health professionals or parents. A preference for peers over family has been identified in past research into help-seeking for substance use problems during adolescence, and may increase with the severity of symptoms [43]. These findings highlight the need for interventions that aim to increase rates of help-seeking for substance use problems specifically, as well as improving general mental health literacy skills.

Just under 60% of the sample correctly identified depression, which is consistent with the results of previous studies examining problem recognition in Australian adolescents [19, 20]. A slightly smaller percentage (52%) correctly identified alcohol misuse, while 9% incorrectly labelled the problems described in the alcohol vignette as another mental health condition, and a further 13% did not know what was wrong or considered the behaviours described to be ‘normal’ or just part of growing up. In contrast, approximately 4% and 7%, of participants gave similar responses to the depression vignette. While overall rates of correct recognition were similar, these results nevertheless suggest that young people may have more difficulty recognising harmful drinking compared to symptoms of depression, and may be more likely to misidentify alcohol misuse as another mental health problem or minimise it as normal adolescent behaviour. Potentially, this could be one influence contributing to the low rates of help-seeking for alcohol misuse compared to depression.

While the majority of participants agreed that the problems described in both vignettes warranted professional help, one-quarter were undecided or disagreed. However, when participants were asked whether they would seek professional help if they experienced problems similar to those described in the vignettes, fewer than half said they were likely to do so. This is surprising, as previous research suggests that 80-90% of adolescents would seek help if they experienced symptoms of depression, with or without alcohol misuse [21, 44]. It is possible that there may be additional perceived barriers to help-seeking for alcohol problems that occur in the absence of other mental health symptoms, such as fear of punishment or concerns about the stigma associated with substance use disorders. Indeed, while participants identified similar barriers to seeking help on the BASH for both type of problems, this measure does not assess concerns about punishment, which may be particularly salient amongst underage drinkers. Similarly, it does not assess concerns about stigmatising responses or judgement on behalf of the counsellor, which may be of greater relevance to alcohol misuse given that substance use disorders have been found to be more severely stigmatised than other mental health conditions [45]. Alternatively, past help-seeking experiences may have influenced adolescents’ future intentions. As health professionals and teachers were the highest rated help sources on helpfulness, yet were still generally considered unhelpful, participants may have been reluctant to seek professional help for future problems based on negative past experiences.

Adolescents reported that they were more likely to encourage their peers to seek help from family members than from other help sources, particularly in regard to depression. The types of formal help that adolescents were most likely to recommend for both types of problem were school counsellors, followed by mental health professionals outside of school. Fewer participants were likely to encourage their peers to seek help from GPs, although they were still recommended by a majority of adolescents. There may be benefits in educating young people about the role of GPs, as there is evidence that many adolescents do not consider them to be an appropriate help source of mental health problems [46, 47], although GPs may also need additional education about what to do when young people approach them [48, 49]. Furthermore, it should be noted that close to 10% of the sample said it was likely that they would not encourage a peer to seek help from anyone.

As expected, females were more likely than males to have sought help in the past, and more likely to have helped a friend seek help. Consistent with the findings of Yap and Jorm [33], they were more likely to rely on professional help than males, and were more likely to report that they would encourage a peer with depression or alcohol misuse to seek help from formal sources. They were also more likely to report they would encourage a peer to seek help from the internet (for depression only) and from family (for alcohol misuse only). A number of unexpected findings emerged, with females significantly more confident that they could help a peer experiencing depressive symptoms seek professional help, but significantly *less* confident that they could talk to a peer experiencing alcohol misuse about their concerns. Females also perceived greater barriers to seeking help for both types of problems, which was surprising given that their help-seeking intentions were stronger and their actual help-seeking behaviours were more frequent than in males. The results suggest that there are likely to be barriers over and above those which were assessed that are more influential during adolescence. Alternatively, females may perceive multiple barriers but are still able to overcome them and seek help. Further research should examine in more detail the factors that discourage help-seeking behaviour amongst females, as well as by problem type.

Teaching adolescents how to better support their peers is essential given that many young people continue to be sought out by friends experiencing mental health problems. Indeed, while one third to one half of participants primarily relied on friends for help, more participants reported seeking help for a friend than for their own problems, which is consistent with previous research [14]. Moreover, despite evidence of poor mental health literacy in regard to problem recognition, the majority of participants were confident that they could approach a peer experiencing symptoms of depression or alcohol misuse, and help them seek professional help. Similar results were reported by Jorm and colleagues [21], who found that adolescents were more confident than young adults despite demonstrating less sophisticated first-aid knowledge. Teaching young people how to help their peers is necessary to ensure that the advice and support given is appropriate, and may also facilitate help-seeking for their own mental health and substance use problems. In the current study, adolescents who agreed that the problems described in the vignettes warranted professional help also reported that they were more likely to seek help if they experienced similar problems. Similarly, adolescents who had helped a peer in the past were more likely to have sought help themselves.

The current study reported baseline data from trial of *MAKINGtheLINK,* a school-based intervention that teaches adolescents how to assist their peers and overcome barriers to accessing professional help. The *MAKINGtheLINK* program was designed to address a number of critical gaps in existing early intervention and health promotion activities by teaching school students how to effectively support their peers and overcome perceived barriers to seeking professional help. As peers become increasingly influential during adolescence, young people are ideally positioned to act as ‘gate-keepers’ to mental health services [50], while equipping them with the knowledge and skills needed to help their peers is likely to facilitate their own help-seeking behaviour. Further analysis of the follow-up data obtained by the *MAKINGtheLINK* trial will provide evidence of the effectiveness of the intervention. If deemed effective, the *MAKINGtheLINK* program will be the first evidence-informed resource that is able to address critical gaps in knowledge and behaviour of adolescents in relation to help-seeking, and could be a valuable resource that is able to be readily implemented by classroom teachers.

There are a number of limitations with this study to consider. First, data collection was at a single point in time, with participants reporting past help-seeking behaviours only, which may have been affected by recall bias or social desirability effects. While help-seeking intentions measured by the GHSQ-V have been found to be correlated with subsequent behaviours [41, 51], longitudinal follow-up is required to determine whether the actual attitudes and intentions reported in the current study influence adolescents’ future help-seeking behaviours. In addition, the gender of the peer described in the vignette was fixed and did not vary according to participant gender. This may have influenced adolescents’ help-seeking intentions in relation to their peers, as recipient gender has been found to influence the mental health first aid actions taken by young people [33]. Finally, the hypothetical situations described in the vignettes may not reflect how adolescents respond to real-life situations. Indeed, Marshall and Dunstan [52] recently demonstrated that Australian adolescents were less likely to correctly identify depression when a more naturalistic form of presentation (film) was used (specifically, 23% gave correct responses compared to 33.8% in an equivalent vignette from a previous study). More generally, concerns have been raised about current mental health literacy instruments, and it has been argued that more psychometrically robust measures could be developed to assess the components of this construct, which encompasses problem recognition as well as knowledge and beliefs about mental disorders which aid their management or prevention [53].

Despite their limitations, the results of the current study provide an important addition to the current knowledge regarding help-seeking for mental health and substance misuse problems in Australian adolescents. They suggest that there is still room for considerable improvement in the mental health literacy of Australian adolescents, particularly in regard to problem recognition and treatment beliefs relating to alcohol misuse. Moreover, they highlight the importance of peer training as a means of facilitating early identification and treatment of mental health problems.
